# Inter-rater agreement of comorbid DSM-IV personality disorders in substance abusers

**DOI:** 10.1186/1471-244X-8-37

**Published:** 2008-05-17

**Authors:** Morten Hesse, Birgitte Thylstrup

**Affiliations:** 1 Centre for Alcohol and Drug Research, University of Aarhus, Købmagergade 26E, 1150 Copenhagen C, Denmark

## Abstract

**Background:**

Little is known about the inter-rater agreement of personality disorders in clinical settings.

**Methods:**

Clinicians rated 75 patients with substance use disorders on the DSM-IV criteria of personality disorders in random order, and on rating scales representing the severity of each.

**Results:**

Convergent validity agreement was moderate (range for r = 0.55, 0.67) for cluster B disorders rated with DSM-IV criteria, and discriminant validity was moderate for eight of the ten personality disorders. Convergent validity of the rating scales was only moderate for antisocial and narcissistic personality disorder.

**Discussion:**

Dimensional ratings may be used in research studies and clinical practice with some caution, and may be collected as one of several sources of information to describe the personality of a patient.

## Background

Personality disorders [PD] represent a major challenge to professionals dealing with substance abusers. PDs complicate treatment for substance use disorders [1-3].

The PD categories and their criteria sets were developed by committees of experts drawing upon their collective clinical experience and insight. Revisions made to the PDs for later revisions of the diagnostic manuals also followed this committee/clinical-experience approach, and some have argued that the committees responsible for the revision have not sufficiently considered the research that has been conducted on the psychometric properties of PDs [4]. But replacing rationally derived descriptions with empirically derived alternatives may not solve the problem for researchers or clinicians working with substance abusers. For instance, a study found that the longterm stability and congruence with observer rating was no better for the empirically derived Five Factor Model than for PDs in a sample of substance abusers [5].

The DSM-IV criteria are viewed as more clear and understandable than the DSM-III-R criteria by both laymen and mental health professionals [6].

In clinical practice, most clinical psychologists use clinical observation and deduction based on behaviour in the clinic and clients narratives [7]. Some researchers also argue for the utility of clinical observations [8], and use clinical observations in studies of the psychometric properties of PDs [9,10], including predictive validity [11]. Other researchers argue for the use of self-report instruments such as the Millon Clinical Multiaxial Inventory [12], or for the use of semi-structured interviews [13], such as the Structured Interview for the DSM-III-R PDs [SIDP-R] [14], or the Structured Clinical Interview for the DSM-IV [SCID-II] [15]. Yet other researchers argue for the integration of information from a range of sources as the gold standard of PD research [16,17]. Reliability research has mostly supported interviews and self-report inventories over clinical diagnoses. But clinical diagnoses have mostly been studied as dichotomous yes/no diagnoses, often with low base-rates of disorder, resulting in very poor power to assess agreement [10].

In the nomenclature, PDs are considered categories. However, categorical approaches have produced a number of problems. Comparisons of different structured interviews have shown that while criteria counts correlate highly, agreement about categorical diagnoses is often poor, simply because many subjects fall on either side of diagnostic cut-offs on different interviews [18]. Thus, even though the actual association of two instruments is about as strong as one can expect to find in the field of psychiatry, categorical diagnoses reduce reliability drastically [19]. An alternative to the categorical approach is to see personality disorders as dimensions, rather than categories.

A study on alcohol dependent patients using the International Diagnostic Checklists for Personality Disorders after longterm clinical observation have noted high interrater agreement of paranoid, antisocial, borderline, histrionic, narcissistic, avoidant, dependent and passive-aggressive personality disorders [11]. A limitation to that study was the requirement of three months of abstinence in treatment before diagnoses could be conducted. Requiring three months of abstinence can help raters avoid misinterpretation of temporary withdrawal-related dysfunctions as symptoms of personality disorders. But given the pervasive negative impact of personality disorders on retention and outcome, clinicians need to adjust their treatment to the behavioural, cognitive and emotional problems that patients experience at an early stage in treatment, often before patients can even be detoxified.

A study on a mixed sample of abstinent and non-abstinent substance abusers [10] showed that correlations between dimensional ratings of personality disorder severity with rating scales for each personality disorder resulted in moderate correlations for paranoid, schizotypal, antisocial, and borderline personality disorder, and high-moderate discriminant validity was found for all personality disorders except schizoid and obsessive-compulsive personality disorder. A limitation of that study was the exclusive reliance on the rating scales, which may have reduced psychometric properties compared with specific criteria for personality disorders.

A recent study by Bowden-Jones and colleagues showed that clinical diagnosis of personality disorder resulted in substantial underdiagnosis of personality disorders in a sample of drug and alcohol abusers [20]. When requiring clinicians to make diagnoses, some, more common and well-known diagnoses may be over-rated, at the expense of less common diagnoses [13,21].

Interrater agreement in terms of personality disorders can be seen to reflect the reliability of personality disorder ratings: if an observers' rating of a patients' personality disorder is used as an indicator of the presence of that personality disorder, how certain can anyone be that a different observer under similar circumstances would give a similar rating? However, from a different perspective, interrater agreement can actually be seen as reflecting validity: if a patient seems to be paranoid, schizoid, or antisocial to two different clinicians, it can be seen as an indication that the patient actually displays behaviour consistent with the disorder. In this article, we approach interrater agreement statistically as if it were validity (see below for details). That is, we assess not only the magnitude of correlations between related constructs, but also the correlations between unrelated constructs. The rationale was that some patients could in principle receive high ratings on all personality disorder criteria regardless of which personality disorder the criterion belonged to, whereas another patient could receive low ratings on all criteria, regardless of what personality disorder that criterion belonged to. This could result in high interrater correlations, not because of agreement about the specific personality disorder, but because of agreement concerning the presence of personality disorder criteria in general.

### Purpose of the study

The purpose of the study was to assess convergent and discriminant interrater agrement of the rating scales. The sample for the present study does not overlap with the sample used in the previous study [10].

## Methods

A mixed sample of 75 substance abusers and dual diagnosis patients were included in the study. Ten were from methadone maintenance settings, and the remaining 65 were from various drug free treatment settings (i.e., residential or outpatient drug free treatment settings for illicit drug use problems). Most (59) were men, and the mean age was 33.7 years (standard deviation = 6.9).

Raters were clinicians (mostly social workers or addiction counsellors; some psychologists or nurses) who were actively involved in patients' treatment. The rating scales used included ten rating scales with scores ranging from 0 to 100% representing the 10 DSM-IV personality disorders [10], and the 79 criteria for the same personality disorders listed in the DSM-IV manual [22]. The criteria were listed in random order to avoid halo bias [9]. Scoring options were 0 (no indication of presence of criterion), 1 (criterion present, but inconsistently, or not causing impairment), and 2 (criterion present, consistently and causing impairment). Based on these scorings, we produced scales that could range from 0 to 2 times the number of criteria listed for the disorder.

All staff members included in the present analysis had participated in training with at least two days of education on PDs, and had been asked to read through a Danish translation of the criteria for each of the 10 PDs provided by one or both of the authors. In 26 of the ratings there were more than 2 rating each patient. In these cases, two were selected using random numbers. The final number of clinicians included with the ratings were 63. Further, on the first sheet of the form, clinicians indicated what kind of contact they had had with patients (counseling, group counseling, and unstructured millieu observation).

Construct validity was analyzed using traditional indices [23]. Convergent and discriminant validity coefficients were calculated as Pearson correlations. Convergent validity is measured as correlations between the same construct measured with different raters, and discriminant correlations denotes correlations between different constructs measured either by the same rater or by different raters. We also calculated the intraclass correlations coefficients for convergent correlations.

Discriminant correlations that exceed convergent correlations are termed comparison violations. That is, instances where correlations between unrelated constructs exceed correlations between related constructs. For each PD scale 18 same-rater discriminant correlations and 18 inter-rater discriminant correlations were calculated from three 10 by 10 matrices (the matrices containing inter-rater, judge 1, and judge 2 correlations). We report the reliability indexes by clusters, according to the DSM-IV (i.e., cluster A, odd-ecccentric, paranoid, schizoid schizotypal; cluster B, dramatic-erratic, antisocial, borderline, histrionic, narcissistic; and cluster C, anxious-fearful, avoidant, dependent, obsessive-compulsive [22]).

According to the guidelines provided by Bagozzi and Yi [24], discriminant validity is considered high at less than 5% comparison violations, moderate at 5–33%, and low at more than 33%. Convergent validity is considered high at *r *> 0.70, moderate at 0.50–0.70, and minimal at 0.30–0.50 [23]. Power analysis showed that the power to detect moderate convergent validity was acceptable with a sample size of 76 (alpha set to 0.005 to adjust for multiple significance tests, power = 0.97). The power to detect marginal convergent validity was not acceptable, but the scientific and clinical significance of agreement that is only marginal is also limited.

Ethical committees in Denmark do not assess the ethical appropriateness or otherwise of studies regarding procedures except if the study involves invasive procedures or the use of medications. However, to the best of our knowledge, we have not violated the Declaration of Helsinki [25] or other ethical issues in relation to the data collection for this study.

## Results

From all ratings, 79% reported individual counseling contact, 79% reported unstructured millieu observations, and 61% reported having had group counseling contact. Almost half (49%) reported all three kinds of contact. Prevalence of PD, mean scores and standard deviations on each scale can be seen in table 1. Also reported is Cronbach's alpha, a measure of internal consistency for each PD scale.

**Table 1:** Descriptive statistics for the sample

	Prevalence (over cut-off for diagnosis with a score of 2 per criterion)	Cronbach's alpha for criteria scales	Mean score for criteria scales	Standard deviation	Mean score for rating scales	Standard deviation
Paranoid	8%	0.79	5.2	3.1	38.9	20.4
Schizoid	5%	0.73	3.9	2.8	23.4	22.7
Schizotypal	1%	0.79	4.5	3.3	25.3	22.4
Antisocial	12%	0.81	4.8	3.3	37.9	26.9
Borderline	1%	0.72	6.4	3.5	35.3	22.2
Histrionic	3%	0.72	4.4	3.1	23.8	22.5
Narcissistic	5%	0.87	4.7	3.9	33.3	27.3
Avoidant	4%	0.81	4.9	3.1	29.6	23.5
Dependent	5%	0.80	5.9	3.7	30.6	24.4
Obs-Com	0%	0.72	3.8	2.9	31.0	26.0

### Convergent and discriminant validity of DSM-IV criteria

The correlations and percent comparison violations for the DSM-IV criteria can be seen in table 2. In table 2 and 3, convergent correlations (i.e., correlations between two different clinicians' ratings of the same patient on the same construct) and unadjusted probablities of the correlations are shown in column 2 and 3. The total number of comparison violations is shown in column 4 (i.e., proportion of correlations between the construct and any other construct rated by either the same or a different clinican that exceed the convergent correlation). The number of comparison violations by the same rater is shown in column 5 (i.e., the proportion of correlations of the same clinicians' ratings of the same patient on different constructs that exceed the convergent correlation). The number of comparison violations by the different raters is shown in column 6 (i.e., the proportion of correlations between different clinicians' ratings of the same patient on different constructs that exceed the convergent correlation).

**Table 2:** Convergent and discriminant validity of DSM-IV criteria

	Pearson correlations	Unadjusted probability	Total percent comparison violations	Percent same rater comparison violations	Percent different rater comparison violations
Paranoid	0.45	< 0.001	29%	53%	5%
Schizoid	0.26	0.022	37%	37%	37%
Schizotypal	0.43	< 0.001	16%	32%	0%
Antisocial	0.55	< 0.001	18%	32%	5%
Borderline	0.55	< 0.001	21%	42%	0%
Histrionic	0.64	< 0.001	3%	5%	0%
Narcissistic	0.67	< 0.001	5%	11%	0%
Avoidant	0.29	0.008	39%	58%	21%
Dependent	0.43	< 0.001	13%	26%	0%
Obs-Com	0.47	< 0.001	8%	16%	0%

**Table 3:** Convergent and discriminant validity of rating scales representing personality disorders

	Pearson correlations	Unadjusted probability	Total percent comparison violations	Percent same rater comparison violations	Percent different rater comparison violations
Paranoid	0.29	0.015	29%	42%	16%
Schizoid	0.19	0.069	47%	63%	32%
Schizotypal	0.04	0.496	74%	95%	53%
Antisocial	0.65	< 0.001	5%	11%	0%
Borderline	0.26	0.029	42%	68%	16%
Histrionic	0.49	< 0.001	13%	26%	0%
Narcissistic	0.59	< 0.001	5%	11%	0%
Avoidant	0.38	0.001	16%	32%	0%
Dependent	0.01	0.873	71%	95%	47%
Obs-Com	0.47	< 0.001	5%	11%	0%

Agreement was moderate for all cluster B disorders (range: 0.55, 0.67), and minimal or worse for all cluster A and C disorders (range: 0.26, 0.47). Intraclass correlations did not differ substantially from Pearson product-momemt correlations (the highest difference was found for avoidant personality disorder: r = 0.29, ICC = 0.32). Discriminant validity was moderate for paranoid, schizotypal, dependent and obsessive-compulsive personality disorder and all cluster B disorders (range of comparison violations: 5–21%).

By far the largest number of comparison violations were same-rater violations (i.e., correlations between the same rater's rating of the same patient on different personality disorders exceeding different rater's rating of the same patient on the same personality disorder). Only for schizoid and avoidant personality disorders, there was a number of discriminant violations.

### Convergent and discriminant validity of rating scales

The correlations and percent comparison violations for the DSM-IV criteria can be seen in table 3.

Agreement was moderate for antisocial (r = 0.65) and narcissistic personality disorder (0.59), and minimal or worse for all other disorders (range: 0.01, 0.49). Discriminant validity was moderate for paranoid, antisocial, histrionic, narcissistic, avoidant and obsessive-compulsive disorders (range of comparison violations: 5–29%). The intraclasscorrelations did not differ substantially from the Pearson product-moment correlations. The highest difference was found for dependent PD (r = 0.02, ICC = 0.01).

Again, the largest number of comparison violations were same-rater violations (i.e., correlations between the same rater's rating of the same patient on different personality disorders exceeding different rater's rating of the same patient on the same personality disorder). Only for disorders with very weak convergent validity, a number of comparison violations were found (i.e., schizoid, schizotypal and dependent personality disorders).

## Discussion

Clinicians in this sample rated personality disorders based only on their knowledge of patients. Convergent and discriminant correlations were mostly as good as convergent and discriminant correlations based on interview vs. questionnaire conducted with the same patient [26,27], and somewhat better than self/other agreement on personality disorders [28]. When clinician ratings offer a reliable perspective on personality disorders, such observations may add an important perspective to ratings of personality pathology. Based on the current findings, it appears that there is support for the reliability of staff observations of 8 of 10 disorders at the given level of alpha (0.005). The exceptions, schizoid and avoidant personality disorders, are both related to introversion [29] and interpersonal withdrawal [22]. It may not be so easy to identify such traits, especially in clinical contexts where many other patients draw attention to themselves.

The use of dimensional scores increased the power of the present study substantially. Having dichotomized diagnoses would have led to low baserates (with a maximum of 12 percent for antisocial personality disorder), and would therefore almost certainly not have led to reasonable agreement – eventhough the scales representing the same traits showed statistically significant convergent correlations for 8 of 10 personality disorders with the present alpha.

The interrater agreement on personality disorders was better for criterion-based ratings than for simple rating scales. However, the difference was not large. The correlation of raters' rating of personality disorders with another rater in this sample ranged from 0.29 for schizoid personality to a maximum of 0.67 for narcissistic personality disorder. And while convergent validity was improved compared with rating scales, the amount of high discriminant correlations was still substantial. In particular, same-rater correlations were in many cases larger than convergent correlations. Thus, when a clinician rated a patient, he was likely to either give high scores regardless of what personality disorder was rated, or give low scores.

The utility of data sources depend on the purpose of assessment. For certain research purposes, clinical observation may suffice, with the notable exception of schizoid and avoidant traits.

### Limitations

The study has some limitations due to the sample group: The sample is heterogeneous with regard to substance and level of substance intake. Therefore, some of the variability in terms of personality disorder severity may be related to variability in substance-related problems, especially chronic substance-related problems. However, when the patients were rated by clinicians, they were actively involved in treatment, and the most acute substance-related problems that could mimic personality disorders would likely be reduced at this point.

A further limitation is that we did not collect information about the number of contacts or the amount of time spent with patients. We did have some information about the type of contact.

Further studies of interrater agreement of comorbid personality disorders can benefit from including detailed information on level and kind of drug intake as well as conducting the rating at a certain point after beginning of treatment. However, the method used resembles clinical practice, where it is often not clear at the beginning of treatment, what personality disorders a patient has, yet clinicians make observations and judgments about patients' long-standing problems and traits [7].

A further limitation was the absence of self-reported data on personality disorders from a self-report inventory or a semi-structured interview. The study could therefore only rate to what extent two external observers would make the same observations. On the other hand, semi-structured interviews and inventories are susceptible to biases, both in the form of impression management and patients' varying degree of insight into their problems.

## Conclusion

In conclusion, clinicians' ratings of personality disorders are sufficiently reliable to constitute a source of information about patients' personality disorders, with the exception of schizoid and avoidant personality disorder.

## Competing interests

The authors declare that they have no competing interests.

## Authors' contributions

Both authors cooperated to design the study. MH conducted the statistical analyses and made a first draft of the manuscript. Both authors revised and changed the manuscript several times. Both authors read and approved the final manuscript.

## Pre-publication history

The pre-publication history for this paper can be accessed here:


